# Current State and Future Prospects of Direct-to-Consumer Pharmacogenetics

**DOI:** 10.3389/fphar.2012.00152

**Published:** 2012-08-20

**Authors:** E. W. Chua, M. A. Kennedy

**Affiliations:** ^1^Carney Centre for Pharmacogenomics, University of Otago ChristchurchChristchurch, New Zealand; ^2^Faculty of Pharmacy, Universiti Kebangsaan MalaysiaKuala Lumpur, Malaysia

**Keywords:** direct-to-consumer, pharmacogenetics, personal genome, pharmacogenomics, personalized medicine

## Abstract

Direct-to-consumer (DTC) DNA testing has grown from contentious beginnings into a global industry, by providing a wide range of personal genomic information directly to its clients. These companies, typified by the well-established 23andMe, generally carry out a gene-chip analysis of single-nucleotide polymorphisms (SNPs) using DNA extracted from a saliva sample. These genetic data are then assimilated and provided direct to the client, with varying degrees of interpretation. Although much debate has focused on the limitations and ethical aspects of providing genotypes for disease risk alleles, the provision of pharmacogenetic results by DTC companies is less studied. We set out to evaluate current DTC pharmacogenetics offerings, and then to consider how these services might best evolve and adapt in order to play a potentially useful future role in delivery of personalized medicine.

## Introduction

The provision of direct-to-consumer (DTC) genotyping services gives patients access to personal genetic information that is often of uncertain value, and that the majority of medical professionals are not sufficiently confident of handling (Stanek et al., 2012). Despite the contentious beginnings of these services and debates about the value of the information they provide (Platt, 2009; Vashlishan Murray et al., 2010), it is fair to suggest that DTC companies have played an important role in raising public awareness around genetics, empowering individuals to seek more knowledge about their own genomes and enabling them to encourage their doctors to also consider this information. It seems likely these companies will remain a significant force as providers of genome information to the public, and it is conceivable that they will evolve to become major players in the healthcare setting.

Setting aside debates around the value and dangers of genotyping risk alleles for complex disease, we focus here on the *pharmacogenetic* information currently provided by DTC companies, and assess the value and limitations of this information. We have primarily depended on information gleaned from company websites, and we have adopted a rather broad definition of DTC pharmacogenetics in order to encompass a wide range of services. Companies were deemed to be “DTC” if they provide a mechanism for the consumers to directly order the tests advertised online, either with or without prescription by a physician. The list we have developed (available from http://www.otago.ac.nz/christchurch/research/carneycentre/publications/otago033875) is largely a subset of that released by the Genetics and Public Policy Centre (Dvoskin, 2011), with a few additions that we have come across in the review process. The listed companies employ two general approaches, one which is purely DTC and does not involve consultation with independent doctors (23andMe, GenePlanet, Matrix Genomics, Theranostics Lab), and one that does (Genelex, Kimball Genetics, Navigenics, and Pathway Genomics). The list is not exhaustive and is primarily meant to illustrate the current state of DTC pharmacogenetic testing services, although the landscape is changing rapidly.

The types of DTC pharmacogenetic tests offered encompass a wide range, with certain drug-gene pairs being exclusively offered by some companies (Table 1). We have excluded tests that are not strictly related to clinical pharmacogenetics: alcohol consumption, smoking and risk of esophageal cancer, caffeine metabolism, and heroin addiction. In the majority of cases selection or inclusion of pharmacogenes or markers will be constrained by the genotyping platform employed. PCR-based methods allow analysis of a relatively small number of variants, but are very easily customized to incorporate new tests. Chip-based platforms interrogate very large numbers of variants, but tend to be less adaptable due to production costs. Some companies, such as 23andMe, employ a gene-chip genotyping approach that provides genome-wide targeted probing of about one million single-nucleotide polymorphisms (SNPs), and others, such as Genelex, use more readily customizable PCR-based genotyping platforms.

**Table 1:** **Range of DTC pharmacogenetic tests**.

Pharmacogenetic trait	23andMe	Genelex	GenePlanet	Kimball Genetics	Matrix Genomics	Navigenics	Pathway Genomics	Theranostics Lab
**ADVERSE DRUG REACTIONS (ADRS)**								
Abacavir hypersensitivity	HLA-B*5701 rs2395029					HLA-B*5701 rs2395029	Unknown	
Aminoglycoside-induced ototoxicity							Unknown	
Carbamazepine hypersensitivity						HLA-B*1502 rs2844682 rs3909184	Unknown	
Floxacillin-induced hepatotoxicity	HLA-B*5701 rs2395029					HLA-B*5701 rs2395029		
Fluorouracil toxicity	DPYD*2A	*DPYD* allele				DPYD*2A		
Interferon-induced thrombocytopenia	*ITPA/DDRGK1* rs6139030							
Irinotecan toxicity		UGT1A1*28				UGT1A1*28 rs10929302		
Lumiracoxib-induced hepatotoxicity	*HLA alleles* rs 3129900							
Methotrexate toxicity							Unknown	
Oral contraceptives, hormone replacement therapy, and risk of venous thromboembolism (VTE)	Factor V Leiden Prothrombin G20210A variant						Unknown	
Postoperative nausea and vomiting (PONV)	*DRD2 Taq1A*							
Pseudocholinesterase deficiency	*BCHE* variants: Asp70Gly (A) Thr243Met (F1) Gly390Val (F2)					*BCHE* variants: Asp70Gly (A) BCHE*539T		
Ribavirin-induced anemia	*ITPA* variant: Pro32Thr							
Selective serotonin reuptake inhibitors (SSRI) and sexual dysfunction	*HTR2A* variant							
Statin-induced myopathy	SLCO1B1*5 *COQ2* variant					SLCO1B1*5	Unknown	SLCO1B1*5
Thiopurine toxicity						TPMT*2 TPMT*3A, *3B, *3C		
**DRUG EFFICACY**								
Antidepressants	*ABCB1* rs2032583	CYP2D6*1-*15 CYP2D6*17, *41	*ABCB1* rs2032583		*CYP2D6* alleles, CYP2C19*2-*8, CYP2C19*17			
Aspirin and stroke; myocardial infarction (MI)			*ITGB3* variant: Leu33Pro					
Beta-blocker and heart failure	*ADRB1* variant: Arg389Gly		*ADRB1* variant: Arg389Gly			*GRK5* variant: ln41Leu		
Beta-blocker and hypertension								
Clopidogrel and stroke; MI	CYP2C19*2-*4 CYP2C19*8, *17	CYP2C19*2-*8 CYP2C19*17			CYP2C19*2-*8, CYP2C19*17	CYP2C19*2, *3	Unknown	CYP2C19*2, *3 CYP2C19*17
Interferon-α/ribavirin and hepatitis C	*IL28B* rs8099917							
Interferon-β and multiple sclerosis	*GPC5* rs7987675							
Metformin and type 2 diabetes mellitus	*ATM* rs4585							
Naltrexone and alcohol dependence	*OPRM1* variant: Asn40Asp							
Sildenafil and erectile dysfunction			*GNB3* variant: Ser275Ser					
Statins and MI			*KIF6* variant: Trp719Arg			*ApoE2*, *E3*, or *E4* allele	Unknown	
Tamoxifen and breast cancer		CYP2D6*1-*15 CYP2D6*17, *41			*CYP2D6* CYP2C19*17			
Triptans and migraine			*GNB3* variant: Ser275Ser					
Warfarin sensitivity	VKORC1*2 CYP2C9*2, *3	VKORC1*2 CYP2C9*2-*8, *11, *13	VKORC1*2 CYP2C9*2, *3	VKORC1*2 CYP2C9*2, *3	VKORC1*2 CYP2C9*2, *3, *5, *6 CYP4F2*3 *GCX* variant	VKORC1*2 CYP2C9*2, *3 CYP4F2*3	Unknown	VKORC1*2 CYP2C9*2, *3

*Shaded boxes indicate that the tests are not offered by the companies. Pharmacogenetic traits with clear FDA recommendations are underlined. Matrix Genomics also genotypes *CYP3A5*; Genelex also offers tests for *CYP1A2* and *NAT2**.

## Current DTC Pharmacogenetics Offerings

Our review of DTC pharmacogenetic testing services illustrated that current offerings in the pharmacogenomics space are patchy and quite limited. These limitations occur at the level of the specific genes selected for genotyping, which in many cases is governed by the technology platform used (as mentioned above), and in the range of gene variants or alleles that are genotyped. These limitations are important because they can influence interpretation of results and the accuracy of predictions based on the data (Ng et al., 2009). Genotype-based classification of metabolic status can be clouded by the existence of multiple alleles that dictate enzymatic function, especially when assignment of metabolic status depends largely on the presence or absence of loss-of-function alleles. Pitfalls arise when defective alleles that are not typed are, in fact, present (PharmGKB, 2011). For example, about 30 defective *CYP2C19* alleles have been identified to date, and the prevalence of these alleles is ethnicity-dependent. All companies only measure a proportion of the alleles in their *CYP2C19* tests and inclusion of alleles differs between companies (Table 1). This could lead to varying degrees of test accuracy across different ethnic groups. Genetic tests that measure only CYP2C19*2 and *3 alleles would correctly identify approximately 88% of Caucasian poor metabolizers (PMs), but nearly 100% of Oriental PMs. Typing of more defective alleles such as CYP2C19*4 would improve the test accuracy among Caucasians, but have no additional benefits for patients of other ancestries (de Morais et al., 1994; Ferguson et al., 1998; Ibeanu et al., 1998; Sim, 2011). Moreover, other factors such as gene dosage, modifier genes, drug–drug interactions, and relevant environmental effects could also affect the accuracy of phenotype prediction.

The influences of ethnicity are also notable when the allele- or SNP-phenotype association is used to predict the development of an adverse drug reaction (ADR) or response to a drug. This is an area in which current evidence varies considerably across ethnic groups. For instance, HLA-B*1502 allele is a strong predictor of carbamazepine-induced severe cutaneous adverse reactions (SCARs) in Han Chinese patients, but it is absent in other populations such as Caucasians (Chung et al., 2004; McCormack et al., 2011). Another less extreme example is the response to interferon-α/ribavirin which is independently influenced by a number of SNPs near the *IL28B* gene region. Two SNPs, namely rs12979860 and rs8099917, have exhibited the strongest signals in several studies involving patients of different ancestries (Lange and Zeuzem, 2011). rs12979860, however, has been found to be less reliable in Japanese patients (Ito et al., 2011), suggesting that data from genotyping a single SNP may not be sufficiently generalizable across populations – a limitation often acknowledged by DTC companies in their reports. Of the companies surveyed only 23andMe offers the *IL28B* test, using the SNP rs8099917 to classify individuals into various response categories. As noted, many DTC companies are restrained by their chip-based genotyping platforms and currently available evidence of SNP-phenotype association, and are therefore less capable of tuning their tests to account for the ethnic diversity of their global customer base.

The categorization of metabolic capacity is also an imprecise process, complicated by lack of a consensual classification system which takes into account various factors that may influence enzymatic function. The categories of PMs, intermediate (IMs), extensive (EMs), or ultra-metabolizers (UMs) are rarely discrete. Rather, they represent a continuous spectrum of metabolic function with significant inter-group overlap. Phenotypic prediction and classification based on a complex array of *CYP2D6* variants, which give rise to both allele-specific and substrate-specific effects on enzymatic activity, is particularly daunting. Genelex, for instance, employs a multiplex PCR approach to detect *CYP2D6* gene duplication and 17 alleles; the diplotypes are subsequently classified into PM, IM, EM, or UM. However, this seemingly comprehensive test has two important caveats. First, classifying the multitude of *CYP2D6* allelic combinations into just four metabolic categories and applying these universally to CYP2D6 substrates can be rather simplistic. Second, default assignment of functional gene duplication can lead to overestimation of CYP2D6 activity when non-functional or reduced-function alleles are duplicated (Gaedigk et al., 2007a); or when the duplication is a false positive as a result of undetected hybrid alleles (Black et al., 2012). These problems are not specific to DTC companies, as they are an ongoing issue for the entire pharmacogenomics community. For example, although an activity score system has been proposed to account for the nuances in enzymatic activity conferred by different *CYP2D6* diplotypes, thereby producing more refined prediction of metabolic status (Gaedigk et al., 2007b), no consensus has yet been reached with regard to the best classification system (Kirchheiner, 2008), and there is evidence to suggest that the traditional system may perform equally well in certain instances (Lötsch et al., 2009).

The most important issue surrounding DTC pharmacogenetics is a lack of unequivocal clinical value for many of the tests on offer. Marketing pharmacogenetic data which are still debatable could be premature; however, where this is made clear in the report the information provided could be of some educational value. For the purposes of this review we used as a guide to “actionable” tests any recommendations in the US Food and Drug Administration (FDA) drug labels, and guidelines from the Dutch Pharmacogenetics Working Group [DPWG; Swen et al., 2011; PharmGKB, 2012; US Food and Drug Administration (FDA), 2012]. Guidelines developed by the Clinical Pharmacogenetics Implementation Consortium (CPIC) are underway and yet to be completed (Relling and Klein, 2011) therefore these guidelines have not been used in our analysis. FDA-approved drug labels containing information on pharmacogenomics biomarkers were screened in an attempt to identify drug-gene pairs for which pharmacogenetic data may provide an “actionable” outcome. Out of the 112 FDA drug-gene pairs, only 30 are good candidates for DTC testing as they are provided with pharmacogenetics-based recommendations in the product inserts. These recommendations vary from specific dose adjustments, or drug avoidance, to vague dosage guidelines, which typically involve dose reduction of uncertain magnitude (Table 2). Thirty-three drug-gene pairs were considered irrelevant in the DTC setting: 27 are associated with cancer genetics, 5 with congenital disorders, and 1 with determination of HIV-1 strain. For the remaining 49 drug-gene pairs, pharmacogenetics-based recommendations are either absent or irrelevant. Testing for this group of 82 drug-gene pairs is therefore probably of little clinical value.

**Table 2:** **Drug-gene pairs with therapeutic recommendations in FDA drug labels and the Dutch Pharmacogenetics Working Group (DPWG) guidelines**.

Treatment modifications to avoid ADRs	Treatment modifications based on metabolic status		Ambiguous recommendations
**FDA-only**	**FDA-only**	**DPWG-only (cont’d)**	**FDA-only**
Carbamazepine/HLA-B*1502	Celecoxib/*CYP2C9*	Metoprolol/*CYP2D6*	Azathioprine/*TPMT*
Dextromethorphan and quinidine/*CYP2D6*	Clobazam/*CYP2C19*	Nortriptyline/*CYP2D6*	Carisoprodol/*CYP2C19*
Pimozide/*CYP2D6*	Iloperidone/*CYP2D6*	Omeprazole/*CYP2C19*	Cevimeline/*CYP2D6*
Rasburicase/*G6PD*	Tetrabenazine/*CYP2D6*	Oxycodone/*CYP2D6*	Chloroquine/*G6PD*
Thioridazine/*CYP2D6*	Warfarin/*CYP2C9*	Pantoprazole/*CYP2C19*	Clopidogrel/*CYP2C19*
	Warfarin/*VKORC1*	Paroxetine/*CYP2D6*	Clozapine/*CYP2D6*
**DPWG-only**		Phenprocoumon/*CYP2C9*	Flurbiprofen/*CYP2C9*
Estrogen-containing oral contraceptives	**DPWG-only**	Phenprocoumon/*VKORC1*	Fluvoxamine/*CYP2D6*
(OCs)/*Factor V Leiden*	Acenocoumarol/*CYP2C9*	Phenytoin/*CYP2C9*	Irinotecan/*UGT1A1*
Tegafur and uracil/*DPYD*	Acenocoumarol/*VKORC1*	Propafenone/*CYP2D6*	Mercaptopurine/*TPMT*
	Amitriptyline/*CYP2D6*	Risperidone/*CYP2D6*	Phenytoin/HLA-B*1502
**Both FDA and DPWG**	Azathioprine/*TPMT*	Sertraline/*CYP2C19*	Thioguanine/*TPMT*
Abacavir/HLA-B*5701	Clomipramine/*CYP2D6*	Tamoxifen/*CYP2D6*
Capecitabine/*DPYD*	Clopidogrel/*CYP2C19*	Thioguanine/*TPMT*
Fluorouracil/*DPYD*	Doxepin/*CYP2D6*	Tramadol/*CYP2D6*
	Escitalopram/*CYP2C19*	Venlafaxine/*CYP2D6*	
	Esomeprazole/*CYP2C19*	Voriconazole/*CYP2C19*	
	Lansoprazole/*CYP2C19*	Zuclopenthixol/*CYP2D6*	
	Flecainide/*CYP2D6*	
	Haloperidol/*CYP2D6*	**Both FDA and DPWG**	
	Imipramine/*CYP2C19*	Aripriprazole/*CYP2D6*	
	Imipramine/*CYP2D6*	Atomoxetine/*CYP2D6*	
	Irinotecan/*UGT1A1*	Citalopram/*CYP2C19*	
	Mercaptopurine/*TPMT*	Codeine/*CYP2D6*	

Currently for most of the drug-gene pairs listed on the FDA website [US Food and Drug Administration (FDA), 2012], genetic screening is recommended but not required by FDA (PharmGKB, 2012). Only a small proportion of the drug-gene pairs tested by DTC companies are likely to have a significant impact on clinical intervention (Table 1). For many drug-gene pairs, consensus guidelines have yet to be established, as evidenced by little overlap between FDA and DPWG in their lists of useful drug-gene pairs (Table 2). Dosing based on thiopurine methyltransferase (TPMT) activity (testing offered by Navigenics), for example, is somewhat ambiguous. While DPWG provides clear-cut dosage guidelines for poor methylator phenotypes, FDA is still uncertain in this regard [Swen et al., 2011; US Food and Drug Administration (FDA), 2012]. The value of pharmacogenetic data provided by DTC companies is therefore limited by lack of effective guidelines for test interpretation, which is of course, a much wider problem in pharmacogenetics. The approaches taken by the CPIC, and other bodies, should steadily lead to improvements in this situation (Relling and Klein, 2011).

## What is the Future for DTC Pharmacogenetics/Pharmacogenomics?

As we have shown, current DTC pharmacogenetics offerings are significantly limited in scope and utility. That said, it is likely these companies are here to stay and it is worth considering how they may look in the future. Several points are worth considering, the first of which relates to the pace of change in genomics technology. The rapid evolution of next generation sequencing technologies has ushered in an era of “personal genomics” (McGuire et al., 2007). As the cost of generating human genome sequences has plummeted, many companies are gearing up to provide, or are already providing, whole human genome sequences, or the more targeted subset of all exons in the genome (the exome). The first signs of transition by DTC companies to the use of genome sequence information rather than genotyping of specific loci are apparent, and it seems inevitable that others will follow this trend. In September 2011, 23andMe announced a pilot project aimed at providing raw exome data to its existing customers, for a price of USD999 (23andMe, 2011). Transition from chip-based or candidate-gene typing to exome sequencing will provide much more detailed pharmacogenetic data and could allow interrogation of multiple markers to add predictive power. Certainly, it should be possible to provide a fairly complete profile of pharmacogenetic variants with known functional effects, but of course many rare variants of unknown function will also be detected and sorting signals from noise will be a significant problem. In addition, it is still difficult to distil accurate copy number variation (CNV) data from sequencing data (Zhang et al., 2011), and until this becomes a more precise process, it will be difficult to provide accurate information on CNVs in pharmacogenes – with *CYP2D6* as the paradigmatic pharmacogene whose activity is affected by CNV.

A major challenge to the application of whole genome data will be the high load of rare variants apparent in human genomes (Nelson et al., 2012). Genotype-phenotype correlations are imperfect even for common pharmacogenetic variants and interpreting the clinical relevance of rare variants will be crucially important to extract maximum value from personal genomes. Resolving this issue will likely require the application of new bioinformatics- and laboratory-based functional screens, but even with improved methods it is probable that prediction of phenotype based on personal genome data will always carry with it a level of uncertainty.

To apply exome or whole genome sequencing (WGS) in the clinical setting, the standard of its performance must be at a level that is equivalent to other diagnostic tests. In the United States, compliance with Clinical Laboratory Improvement Amendments (CLIA) is considered to be a hallmark of quality for laboratory tests (Frueh et al., 2011; Spencer et al., 2011). Most currently available DTC genetic tests are conducted in laboratories with CLIA or equivalent levels of certification. DTC exome sequencing services should also meet similar regulatory requirements. In order to accommodate the global nature and novel features of the service, modernization of national regulations and establishment of an international certification system have been suggested (Frueh et al., 2011; Hauskeller, 2011). Ensuring the diagnostic validity of DTC results in this way could add confidence for the consumer and may position DTC testing companies for a more expansive role in the healthcare setting.

Many critics of DTC genetic testing focus on the reduced role for medical professionals as gatekeepers to complex genetic information (Lenzer and Brownlee, 2008; Anonymous, 2008). However, health care professionals are generally ill-prepared for incorporating pharmacogenetics into their practice (Stanek et al., 2012). It is likely that the activities of DTC companies are educating consumers about genetics and pharmacogenetics, and these consumers will in turn indirectly influence and educate their physicians. Indeed, the models for genome data collection, interpretation, and dissemination adopted by DTC companies, particularly their use of web-based tools for providing continually updated information to clients, can be seen as key components of genomics-based health care (Platt, 2009) and may offer a viable route for the integration of genomic and pharmacogenomic data into more traditional health care structures.

Few health systems are likely to adopt routine, widespread pharmacogenomic testing in the near future. This may provide the opportunity for DTC pharmacogenetics companies to flourish. While many arguments in pharmacogenetics center on the cost-benefit analysis of tests, and the mechanisms for reimbursing testing costs, DTC genetics may help to resolve these debates. It has even been speculated that the affordability of WGS would rapidly improve to a point where genotyping could be regarded as essentially free (Altman, 2011). When patients turn up in a clinic with a full pharmacogenetics profile, obtained from a DTC company and performed in a CLIA-certified laboratory, surely the clinician ought to consider integrating this knowledge in their treatment plans? The obvious caveats on this scenario, which are relatively specific to the DTC companies, include the danger of overstating the value of such tests, and the need to follow clinical guidelines or rely on intelligent and thorough assessments of the evidence base, wherever possible.

## Conclusion

The development of DTC genetic testing has caused considerable controversy, particularly with regard to genes that influence risk of disease. Although perhaps less controversial, the pharmacogenetics offerings of DTC companies also have problems, although the worst of these are precisely those which challenge all practitioners in this field. Two primary problems are the quality of evidence that links genetic variants to functional effects, and the clinical utility of genotypes for specific genes. We anticipate that both of these issues will gradually resolve to a point at which most common, clinically useful gene variants have been identified and incorporated into treatment guidelines. The high level of rare variants observed in human genomes will remain an issue (Nelson et al., 2012), and collectively these may represent a significant pharmacogenetic load in the population that will be more difficult to understand.

Despite their controversial origins DTC companies deserve credit for at least two things. The first is that their activities have raised public awareness of genome analysis and its relevance to therapeutic drug responses. The second is that these companies offer a route to bringing pharmacogenomic data to the table, empowering patients who may feel the need to obtain such data, and enabling them to discuss it with their clinician – which at a minimum may be a useful, two-way learning opportunity. In this situation the patient is taking on a more active role by “initiating” testing and educating and encouraging the physician to consider and perhaps act on this information.

Relatively inflexible genotyping technologies, at present, constrain the range of pharmacogenetic data that is generated, and our survey revealed that the current DTC pharmacogenetics test offerings are patchy and of limited clinical value. However, it is likely these companies will be early adopters of exome and whole genome services, and already we are seeing transition to these kinds of datasets for clients. The companies active in this space are experienced at providing genomic information to their clients via web interfaces in an appealing, understandable, and secure format which is well suited to the ongoing updating of interpretive material – an essential need in a field where the evidence base is continually growing and changing. This may well prove to be an edge that allows such companies to compete effectively in the provision of whole genome or exome data not only in a DTC environment, but also in the clinical setting. The next few years may see some significant changes in traditional clinical laboratory testing models, with DTC companies offering a relatively low cost approach to pharmacogenomic data provision. Over this period most companies are also likely to transition to exome or whole genome methods for generating personal genome data, with a corresponding increase in the range and value of pharmacogenomic data provided. It will be of great interest to see how this innovative and oft maligned industry evolves and grows, and to see what impact this has on the understanding and routine use of pharmacogenomic knowledge.

## Conflict of Interest Statement

The authors declare that the research was conducted in the absence of any commercial or financial relationships that could be construed as a potential conflict of interest.
